# Anti-Inflammatory Effect of Qingpeng Ointment in Atopic Dermatitis-Like Murine Model

**DOI:** 10.1155/2013/907016

**Published:** 2013-08-21

**Authors:** Yun-Zhu Li, Xue-Yan Lu, Wei Jiang, Lin-Feng Li

**Affiliations:** Department of Dermatology, Peking University Third Hospital, 49 North Garden Road, Haidian District, Beijing 100191, China

## Abstract

Qingpeng ointment (QP) is a Chinese medicine which has been used in treatment of atopic dermatitis (AD) in China. AD-like lesions were induced in BALB/c mice by repeated application of 2,4-dinitrofluorobenzene (DNFB) on shaved backs. The mice were then treated for 2 weeks with QP of different concentrations and Mometasone Furoate cream (MF), respectively. Macroscopic and microscopic changes of the skin lesions were observed after the treatment. The levels of serum immunoglobulin (Ig) E, tissue interferon (IFN)-**γ**, and interleukin (IL)-4 and IL-17A and the levels of involucrin, filaggrin, and kallikrein7 in epidermis were measured. The results show severe dermatitis with immune profiles similar to human acute AD. A significant infiltration of CD4^+^ T and mast cells was observed in dermis of lesion but inhibited by QP after a 2-week treatment with it. The production of IgE, IL-4 and the mRNA expression of IL-17A were also suppressed, but the level of IFN-**γ** was increased. MF suppressed all production of these cytokines and IgE. Accordingly, the mechanism of QP on AD might correlate with its ability of modulating the immune dysfunctions rather than suppressing them. It had no effect on expressions of involucrin and filaggrin, except that its vehicle decreased the level of kallikrein7.

## 1. Introduction

Atopic dermatitis (AD), also known as atopic eczema, is a common inflammatory skin disease. The pathogenesis of AD has been attributed largely to abnormalities in the adaptive immune system. The dysfunction of T helper (Th) cells and IgE production may play key roles on it [1–3]. The haptens such as 2,4-dinitrofluorobenzene (DNFB) are used to induce model of murine contact hypersensitivity (MCH). In certain conditions, MCH can generate AD-like lesions and immune responses with Th1 and/or Th2 type inflammation [4]. Therefore, it is often used to study drug mechanism on AD [5–13]. In this work, we repeated applying DNFB as a hapten to induce AD-like lesions in BALB/c mice for research of drug mechanism on AD.

Qingpeng ointment (QP) is a traditional Chinese medicine which has been used in treatment of AD in China. Clinical studies including a multicentered, randomized, double-blind, placebo-controlled study had shown that QP is effective in the treatment of eczema [14]. However, the mechanism of its action is still unclear. In this study, the inflammation of the lesion was observed macroscopically and microscopically to research the anti-inflammatory effect of QP. Skin barrier function is recently considered to play important roles in the pathogenesis of AD. The skin barrier related factors, including involucrin, filaggrin, and kallikrein7, take part in the procedure of skin keratinocyte proliferation and desquamation and they have been found to have significant abnormalities in AD patients [15–19]. These factors were also checked for evaluating the effect of QP on skin barrier function in this research.

Recent years, IL-17 (IL-17A), a new found cytokine, is reported probably playing a negative role in human AD [20]. In this work, the effect of QP treatment on expression of IL-17A was investigated. In addition, the Th1 type cytokine IFN-*γ*, Th2 type cytokine IL-4, and serum IgE were also analyzed to study its possible mechanisms on AD. As topical glucocorticosteroid agents were considered the first used medicine on AD in clinic, Mometasone Furoate cream (MF) treated mice were here used as positive controls in this research.

## 2. Methods and Materials

### 2.1. Animals

Female 8-week-old BALB/c mice (Vital River, China) were maintained under SPF conditions. Animals were housed in an air-conditioned animal room with a constant temperature of 23 ± 2°C and a relative humidity of 40 ± 5%. A standard diet and water were provided by the lab. The study was approved by the Institutional Animal Care and Use Committee of Peking University and experiments were conducted in accordance with the guidelines issued by the animal committee of Peking University Health Science Center for the care and use of laboratory animals.

### 2.2. Induction of Dermatitis and Experimental Schedule

The DNFB (Wako, Japan) was diluted in the mixture of acetone and olive oil (4 : 1). Then, the solution of DNFB (100 *μ*L of 0.5% DNFB) was applied to the shaved backs of mice in the first week for sensitization. After that, 100 *μ*L of 0.2% DNFB was applied twice a week for a further 4 weeks to develop lesions. The model was established at the end of 5th week. During the following experiments, the skin lesions would be re-challenged once one week later to maintain the inflammation. The normal group was applied with nothing during the whole procedure of experiment.

All mice were randomly divided into 7 groups (*n* = 18 for each group) including a normal group, a remained untreated group (model group), and 5 experimental groups. The experimental groups were then treated, respectively, with vehicle of QP, 50% QP in vehicle, 75% QP in vehicle, 100% QP, and Mometasone Furoate cream (MF) (ELOSON, Merck Sharp & Dohme, USA) for 2 weeks after the model was established. The executor had not been told the group names and drug names and the procedure of grouping and treatment with medicines were done in a blind miner. QP (Qingpeng Ruangao) and its vehicle were provided by Cheezheng Tibetan Medicine Company (Gansu, China) and its contents were given in Table 1. Analyses by liquid chromatography and gas chromatography-mass spectrometry at a laboratory in Fudan University (Shanghai, China) showed that no corticosteroid existed in this medicine (data not shown).

All animals were sacrificed at 24 h after the last treatment. Serum was collected and dorsal skin on fixed position of mice was biopsied for histopathology analysis and measurement of tissue cytokines levels.

### 2.3. Thickness of Skin

Thickness of dorsal skin was measured with a micrometer (Hautine International Co., China) before killed. For each mouse, 3 different sites of the back skin were measured randomly, and an average data was taken.

### 2.4. Evaluation of the Skin Lesions

Skin status of each group was photographed before and after the treatment. Skin lesions such as (1) erythem, (2) edema, and (3) scaling were scored as 0 (none), 1 (mild), 2 (moderate), and 3 (severe) as previous study reported [21]. The executor had not been told the group names and the procedure was done in a blind miner.

### 2.5. Histopathology Analysis

The inflammation of the dorsal skin was observed macroscopically and photographed before the mice were sacrificed. The paraffin sections of dorsal skin were dyed with hematoxylin-eosin staining (HE) and were observed under the microscope field of ×100 (Nikon E600).

### 2.6. CD4^+^ T and Mast Cell Counts

The paraffin sections of dorsal skin were analyzed with immunohistology to investigate the effect of QP on CD4^+^ T cell. The primary antibody for CD4^+^ T cell (BS1617) was purchased from Bioworld Technology (USA). The secondary antibody (PV-6001) and DAB stain were purchased from Zhongshan Golden Bridge Biotechnology Company (Beijing, China). The stained sections were observed under the microscope field of ×200 (Nikon E600). Average CD4^+^ T cell numbers in skin were measured by counting 5 different areas in each slide of skin (×200).

Mast cells of dermis were stained with toluidine blue and the section were observed under the microscope field of ×100 (Nikon E600). Average mast cell numbers were measured by counting 5 different areas in each slide of skin (×100).

### 2.7. Enzyme-Linked Immunosorbent Assay (ELISA)

Levels of IL-4 and IFN-*γ* in the skin tissue and serum IgE level were measured with Enzyme-Linked Immunosorbent Assay (ELISA) kits (Dakewei Bio, China) according to the manufacturer's instruction.

### 2.8. RNA Isolation and Quantitative Real-Time Polymerase Chain Reaction

Total RNA from dorsal skin was extracted with Trizol (TransGen Bio, China), and then the RNA concentration was measured with ultraviolet spectrophotometer (Thermo, Germen). According to the manufacturer's protocol, separated total RNA was reverse transcribed into complementary DNA (cDNA) with EasyScript Frist-Strand cDNA Synthesis SuperMix (TransGen Bio, China). After cDNA samples were diluted 20 times with distilled water, we mixed cDNA sample, primers (Table 2), distilled water, and the SYBR Premix Ex Taq (TaKaRa Bio, Dalian) into a 20 *μ*L reaction system. The quantitative real-time PCR was performed in iQ5 real-time PCR system (Bio Rad, USA). The *β*-actin gene was used as an endogenous control to normalize the mRNA expression of IL-17A. Primers for IL-17A and *β*-actin synthesised by Sagon Bio of China were according to previous researches [22]. The results of PCR were calculated and analyzed with method of relative quantification [23]. The results of CT values were transferred into 2^−ΔΔCT^.

### 2.9. Skin Keratinocyte Proliferation and Desquamation-Related Proteins

The expressions of involucrin, filaggrin, and kallikrein7 in epidermis were measured with method of immunohistology. The primary antibodies for involucrin (ad28057) and filaggrin (ad24584) were both purchased from Abcam Company (UK). The primary antibody for kallikrein7 (sc-20381) was purchased from Santa Cruz company (USA). The secondary antibodies (PV-6001, PV-6002, and PV-9003) and DAB stain were all purchased from Zhongshan Golden Bridge Biotechnology company (Beijing, China). The dyed sections were observed under the microscope field of ×100 (Nikon E600). The mean values of optical density (OD) of stained epidermis of each mouse were measured and analyzed with software of Image-Pro Plus 6.0.

### 2.10. Statistical Analysis

The statistical analysis was performed with SPSS of version 16.0. All data that followed a normal distribution was tested with *T* test or Least Significant difference (LSD) test of one way ANOVA. A *P* value less than 0.05 was considered significant.

## 3. Results

### 3.1. Skin Inflammation and Histological Analysis

**Figure 1:**
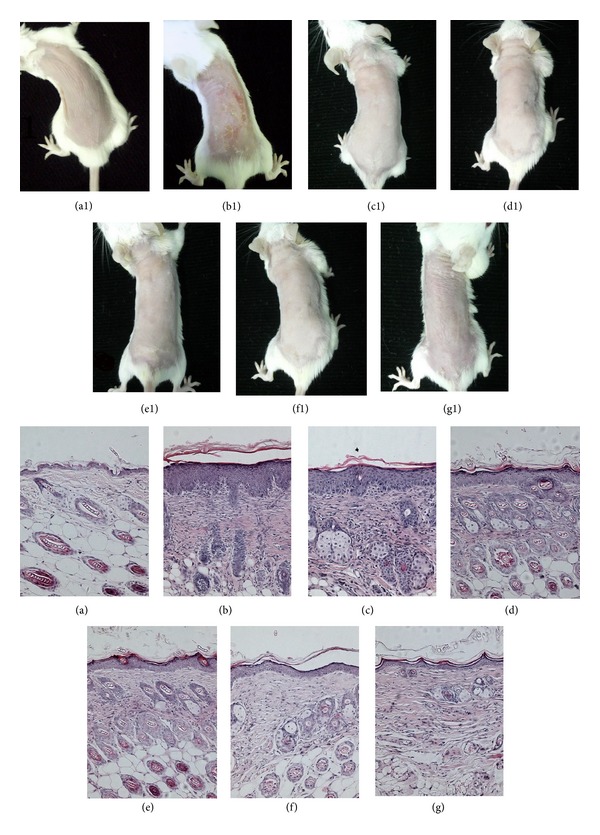
Comparisons of AD-like skin lesions in BALB/c mice after a 2-week treatment. (a1)  Normal mouse; (b1)  model mouse; (c1)   mouse treated with vehicle; (d1)  50% QP-treated mouse; (e1)  75% QP-treated mouse; (f1)  100% QP-treated mouse; (g1)  MF-treated mouse. (a)–(g) were histopathology of corresponding skin. Significant erythema, desquamation, and crusting could be seen on the dorsal skin of model group and lessened significantly in all treated groups. Thickening of the epidermis and inflammatory cell accumulation could be seen in model group but was relieved in QP-treated groups in a dose-related pattern. QP: Qingpeng ointment; MF: Mometasone Furoate cream.

The inflammation of model group was more obvious than other groups with severe erythema, desquamation, and crusting. The inflammation of all treatment groups was decreased. Similar to MF, the dorsal skin of 100% QP treated group was almost normal. The histopathology of skin lesions showed thickening of the epidermis and inflammatory cell accumulation in model group, but QP administration clearly inhibited DNFB-induced inflammation in a dose-related pattern (Figure 1) (Table 3).

### 3.2. CD4^+^ T and Mast Cell Counts

**Figure 2:**
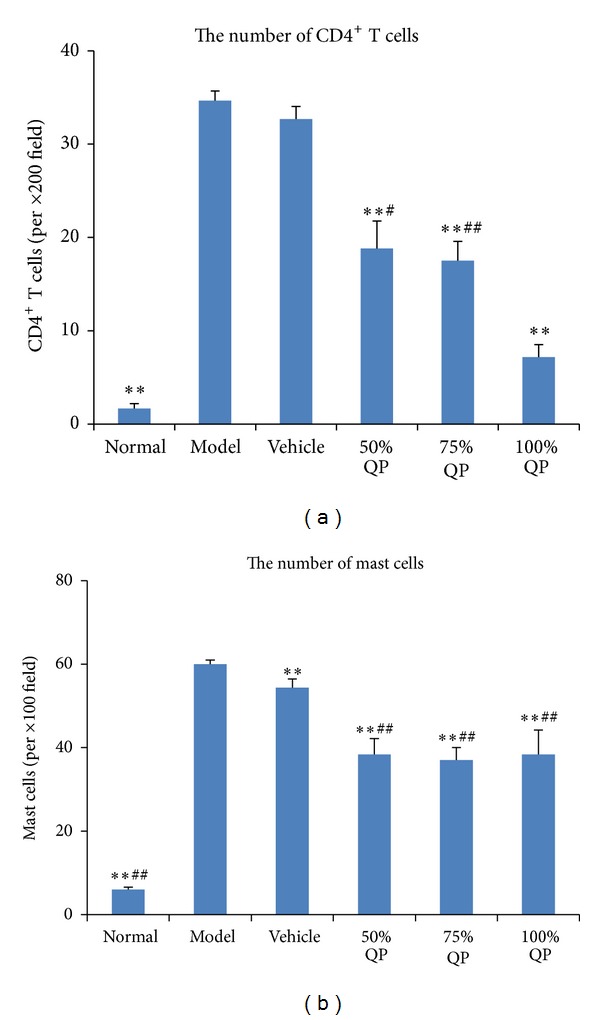
Comparisons of CD4^+^ T cell counts (×200) and mast cell counts (×100) with the treatment of QP. (a) The number of CD4^+^T cells; (b) the number of mast cells.   **P < 0.01 versus model group. QP: Qingpeng ointment. ^#^ *P* < 0.05, ^##^ *P* < 0.01 versus QP vehicle.

CD4^+^ T cells in dermis accumulated in model group, but QP administration clearly inhibited the accumulation in a dose-related pattern. Compared with normal group, mast cell counts were also increased in model group (*P* < 0.01), whereas they declined in 50%, 75%, and 100% QP treated groups compared with vehicle treated group and model group. But there were no significant differences between 50%, 75%, and 100% QP treated groups (Figure 2).

### 3.3. Enzyme-Linked Immunosorbent Assay

**Figure 3:**
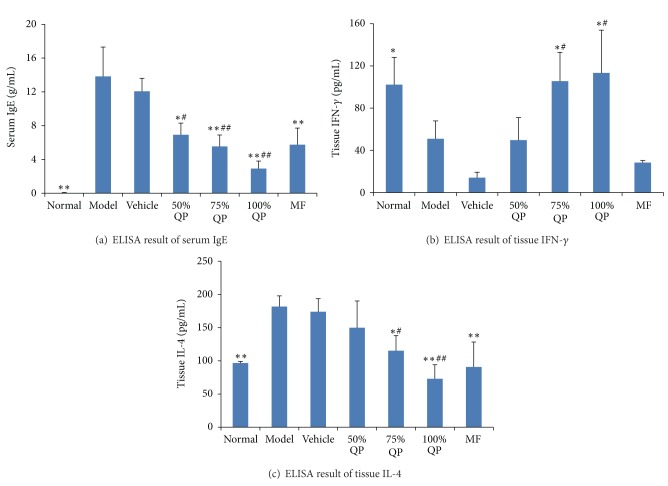
Comparisons of expression of serum IgE, tissue IFN-*γ*, and IL-4. (a) ELISA result of serum IgE; (b) ELISA result of IFN-*γ*; (c) ELISA result of IL-4. *P < 0.05, **P < 0.01 versus model group. QP: Qingpeng ointment; MF: Mometasone Furoate cream. ^#^ *P* < 0.05, ^##^ *P* < 0.01 versus QP vehicle.

The level of serum IgE was significantly elevated in model group, but decreased gradually in QP treated groups (50%, 75%, and 100% QP treated groups) with the elevation of drug concentration. Serum IgE of MF treated group was lower than model group but higher than 100% QP treated group (*P* < 0.01) (Figure 3(a)).

The level of IFN-*γ* of skin tissue in model group decreased significantly (*P* < 0.01), while that in QP treated groups increased gradually along with elevation of drug concentration. The levels of IgE and IFN-*γ* changed significantly between 100% QP group and model group (*P* < 0.05) (Figures 3(a) and 3(b)).

The level of IL-4 in 100% QP treated group was lower than that of model group (*P* < 0.01) but almost similar to normal group and MF treated group (*P* > 0.05). The IL-4 level of QP treated groups decreased gradually with elevation of drug concentration (Figure 3(c)).

### 3.4. The mRNA Expression of IL-17A

**Figure 4:**
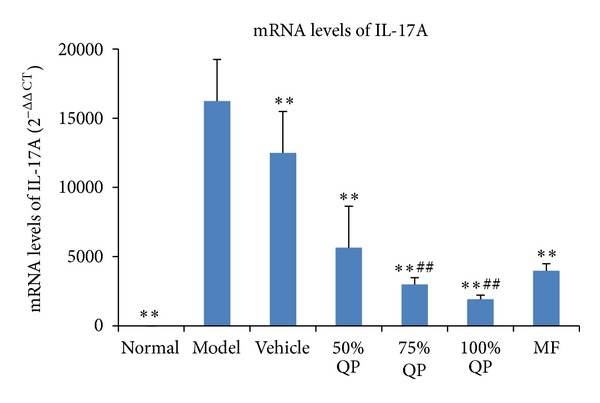
The changes of mRNA levels of IL-17A. The primary value of CT was transferred into 2^−ΔΔCT^. **P < 0.01 versus model group. MF: Mometasone Furoate cream; QP: Qingpeng ointment. ^##^ *P* < 0.01 versus QP vehicle.

The result of quantitative real-time PCR shows that mRNA level of IL-17A increased in model group but decreased after QP administration (*P* < 0.01). MF can also decrease the IL-17A expression (*P* < 0.05) on DNFB-induced skin lesion (Figure 4).

### 3.5. Expressions of Involucrin, Filaggrin, and Kallikrein7

Compared with normal skin, the expressions of involucrin and filaggrin of dermis in model group were decreased, whereas kallikrein7 was increased. QP treatment in this study showed no significant effects in expressions of involucrin and filaggrin. Although level of kallikrein7 was decreased by vehicle and 50%, 75%, and 100% QP, there was no significant difference between vehicle and QP treated groups (Table 4).

## 4. Discussion

AD is a chronic recurrent inflammatory skin disease. Dysregulation of function of Th cells and production of IgE are considered the most important factors in the pathogenesis of AD [2, 3]. Th cells are belonged to CD4^+^ T lymphocytes which include Th1 cells and Th2 cells. The division is based on the pattern of cytokines they secrete. The Th1 is characterized mainly by production of IFN-*γ*, IL-2, and so forth, whereas the Th2 typically synthesizes IL-4, IL-5, and so forth [24]. These distinct cytokine patterns are associated with specific functions. In particular, some cytokines are not restricted to a specific Th cell subtype such as IL-2 [24]. In this work, we applied DNFB as a hapten to BALB/c mice for 5 weeks and the induced dermatitis showed an immune profile of Th2-dominated inflammation which is similar to early reaction of AD with high level of IgE and Th2 cytokines and low level of Th1 cytokines [25, 26].

QP has been used in China for treatment of AD for many years but little is known about its mechanism. A previous study on QP with methods of ELISA by Yuan-Yuan and Lin-Feng [27] had demonstrated that 100% QP and 75% QP significantly decrease the levels of IL-5 after 2-weeks treatment in DNFB-induced AD-like lesions. In addition, the 100% QP elevated the levels of TNF-*α* in skin (*P* < 0.05), which had an unclear relation with mechanism of AD [28, 29]. Resulted from that study, QP showed no effect on IL-1*β* (produced mainly by macrophagocyte) and IL-2 expression in skin (Table 5). In our research, QP administration significantly inhibited the skin inflammation and the production of IgE and IL-4 as MF, but increased the level of tissue IFN-*γ* unlike MF. IL-4 and IL-5 are known to be produced by Th2 cells and they can stimulate B cells to differentiate and secret IgE, whereas IFN-*γ*, secreted mainly by Th1 cell and natural killer (NK) cell, has abilities of activating the neutrophil, NK cell, and Th1 cell and inhibiting the activation of Th2 cell. These results suggested that topical QP might have modulation effects on Th1/Th2 immune deregulations rather than corticosteroids which inhibited the common immune reaction. But as the IFN-*γ* is related to chronic inflammation, the mechanism of QP on chronic lesion still needs to be researched.

Th17 is a new subtype of CD4^+^ T cells which was firstly found that it could secrete IL-17A. Recent researches show that IL-17A can be secreted by other innate cells including CD8^+^ T cells, *γδ* T cells and NKT cells [30]. It is found to have pro-inflammatory functions in host defense against extracellular bacteria, fungi, and possibly some viral infections and cancers [31, 32]. Meanwhile, it can also promote inflammation associated with autoimmunity [33, 34]. Besides AD, IL-17A is also reported higher in other skin diseases [35, 36]. Both MF and QP administrations inhibited the mRNA expression in skin lesion which indicates that QP may be also used on treatment of other IL-17A involved skin diseases.

Besides lymphocytes, mast cells are also demonstrated to be involved in the pathogenesis of atopic dermatitis [37, 38]. Intensive degranulation of mast cells is often observed along with the recruitment of these cells in the inflammatory skin region in AD [39]. Moreover, mast cells activation has been shown to be correlated with the severity of AD [40, 41]. In our study, the number of mast cells increased in induced lesions, whereas QP administration suppressed its accumulation after 2-week treatment. However, the mechanism of this phenomenon needed to be deeply investigated.

More and more studies demonstrated that inherited and/or acquired skin barrier function abnormalities are correlated with the pathogenesis of AD [26, 42]. Involucrin and filaggrin are involved in keratinocyte differentiation while kallikrein7 is a desquamation related enzyme. They all contribute to the permeability barrier function of skin. The extent of its abnormality parallels the severity of the disease phenotype in AD [42]. Our mouse model of AD-like dermatitis showed declined levels of involucrin and filaggrin but an increased level of kallikrein7 which are similar to human AD [17, 19]. But QP treatment on induced AD-like lesions showed little effect on expressions of them in our study except the vehicle of QP which showed a possible ability of suppressing the expression of kallikrein7.

Accordingly, besides its effects on IL-5 and TNF-*α*, the probable mechanism of QP on treatment of AD might also correlate with its functions of inhibiting the infiltration of CD4^+^ T cells and mast cells, reducing expressions of IL-4 and IgE, mRNA expression of IL-17A, and increasing the level of IFN-*γ* on induced Th2-type inflammation in mice.

## 5. Conclusions

Traditional Chinese medicine has been used in the treatment of eczema for a long history in China. In this work, the pathology and molecular biology analyses showed that QP administration inhibited DNFB-induced AD-like dermatitis and infiltration of CD4^+^ T cells and mast cells on lesions of BALB/c mice. The levels of IgE, IL-4, and IL-17A were decreased as MF, but the level of IFN-*γ* was increased which was different from MF. This study therefore provides biological evidence supporting the use of QP as an alternative medicine for the treatment of AD. However, more studies especially on the detailed pharmacy of its elements are still needed to explore its mechanism further.

## Figures and Tables

**Table 1:** Ingredients of Qingpeng ointment (Cheezheng*).

Name	Dose (g)
*Oxytropis falcata Bunge *	100
*Rheum lhasaense *	50
*Aconitum pendulum Busch *	75
*Chebulae Fructus* (without core)	100
*Terminaliae Belliricae Fructus *	100
*Phyllanthi Fructus *	100
*Benzoinum *	35
*Tinospora sinensis *	150
Muscone	25
Vehicle (liquid paraffin, glycerol, emulsifier, water, etc.)	To 5000

*Standard number: Guo Jia Yao Pin Biao Zhun WS3-BC-0319-95-2009; license number: Guo Yao Zhun Zi Z54020140; quality was examined according to Pharmacopoeia of the People's Republic of China (Chinese Pharmacopoeia), Edition 2005, Part I, Appendices I R, VI B, and D.

**Table 2:** Primers of quantitative real-time polymerase chain reaction.

Gene symbol	Primers	GC (%)	*T*_*m*_ (°C)
*β*-actin	F: 5′-GCT TCT TTG CAG CTC CTT CGT	52.3	59.8
	R: 5′-AGC GCA GCG ATA TCG TCA TC	55	62
IL-17A	F: 5′-CTC ACC CGT TCC ACG TCA CCC T	59.1	63.8
	R: 5′-CCA GCT TTC CCT CCG CAT T	57.9	59.7

**Table 3:** Evaluation of the skin lesions and the thickness of skin.

	Score of the skin lesions	Thickness of skin (mm)
Normal	0 ± 0**	0.35 ± 0.06**
Model	8.33 ± 0.52	1.07 ± 0.18
Vehicle	7.83 ± 0.41	1.06 ± 0.07
50% QP	5.50 ± 1.05**	0.95 ± 0.03*
75% QP	3.17 ± 0.75**	0.87 ± 0.08**
100% QP	0.50 ± 0.55**	0.44 ± 0.06**
MF	0.67 ± 0.52**	0.43 ± 0.01**

Values are presented as mean ± SEM. *P < 0.05, **P < 0.01 versus model group. QP: Qingpeng ointment; MF: Mometasone Furoate cream. Skin lesions such as (1) erythema, (2) edema, and (3) scaling were scored as 0 (none), 1 (mild), 2 (moderate), and 3 (severe). Thickness of skin was randomly measured with 3 different sites of the back. Total data was calculated for each mouse, and the average values were taken to be analyzed.

**Table 4:** OD values of involucrin, filaggrin, and kallikrein7 in epidermis.

Groups	Involucrin	Filaggrin	Kallikrein7
Normal	0.55 ± 0.05^∗#^	0.71 ± 0.08^∗∗##^	0.42 ± 0.02*
Model	0.52 ± 0.01	0.54 ± 0.03	0.44 ± 0.02^#^
Vehicle	0.50 ± 0.01	0.56 ± 0.03	0.41 ± 0.02*
50% QP	0.49 ± 0.02	0.57 ± 0.04	0.41 ± 0.01**
75% QP	0.48 ± 0.03	0.58 ± 0.02	0.41 ± 0.01**
100% QP	0.47 ± 0.02	0.58 ± 0.02	0.40 ± 0.02**

Values are presented as mean ± SEM. *P < 0.05, **P < 0.01 versus model group; ^#^ *P* < 0.05, ^##^ *P* < 0.01 versus vehicle group. QP: Qingpeng ointment; MF: Mometasone Furoate cream.

**Table 5:** Changes of IL-1*β*, IL-2, TNF-*α*, and IL-5 levels in skin (pg/mL).

Groups	IL-1*β*	IL-2	TNF-*α*	IL-5
Normal	7.94 ± 0.41^∗#^	7.34 ± 0.39^∗#^	14.67 ± 0.74^∗#^	7.56 ± 0.78^∗#^
Model	5.39 ± 0.68	6.62 ± 0.66	9.38 ± 0.82	22.62 ± 1.90
Vehicle	5.30 ± 0.82	6.58 ± 0.61	11.23 ± 0.51*	18.43 ± 1.19*
50% QP	5.28 ± 0.40	6.54 ± 0.24	11.19 ± 0.29*	17.78 ± 0.70*
75% QP	5.44 ± 0.60	6.56 ± 0.19	11.33 ± 0.37*	16.82 ± 0.59^∗#^
100% QP	5.50 ± 0.72	6.60 ± 0.79	11.80 ± 0.39^∗#^	14.23 ± 1.36^∗#^

Values are presented as mean ± SEM. *P < 0.05, versus model group, ^#^ *P* < 0.05, versus vehicle group. QP: Qingpeng ointment; MF: Mometasone Furoate cream.

## References

[1] Bonness S, Bieber T (2007). Molecular basis of atopic dermatitis. *Current Opinion in Allergy and Clinical Immunology*.

[2] Novak N (2009). New insights into the mechanism and management of allergic diseases: atopic dermatitis. *Allergy*.

[3] Verhagen J, Akdis M, Traidl-Hoffmann C (2006). Absence of T-regulatory cell expression and function in atopic dermatitis skin. *Journal of Allergy and Clinical Immunology*.

[4] Honda T, Egawa G, Grabbe S, Kabashima K (2013). Update of immune events in the murine contact hypersensitivity model: toward the understanding of allergic contact dermatitis. *Journal of Investigative Dermatology*.

[5] Matsuda H, Watanabe N, Geba GP (1997). Development of atopic dermatitis-like skin lesion with IgE hyperproduction in NC/Nga mice. *International Immunology*.

[6] Gao XK, Nakamura N, Fuseda K, Tanaka H, Inagaki N, Nagai H (2004). Establishment of allergic dermatitis in NC/Nga mice as a model for severe atopic dermatitis. *Biological and Pharmaceutical Bulletin*.

[7] Tomimori Y, Tanaka Y, Goto M, Fukuda Y (2005). Repeated topical challenge with chemical antigen elicits sustained dermatitis in NC/Nga mice in specific-pathogen-free condition. *Journal of Investigative Dermatology*.

[8] Jin H, He R, Oyoshi M, Geha RS (2009). Animal models of atopic dermatitis. *The Journal of Investigative Dermatology*.

[9] Inagaki N, Nagai H (2009). Analysis of the mechanism for the development of allergic skin inflammation and the application for its treatment: mouse models for the development of remedies for human allergic dermatitis. *Journal of Pharmacological Sciences*.

[10] Jung BG, Cho SJ, Koh HB, Han DU, Lee BJ (2010). Fermented Maesil (*Prunus mume*) with probiotics inhibits development of atopic dermatitis-like skin lesions in NC/Nga mice. *Veterinary Dermatology*.

[11] Choi MS, Kim EC, Lee HS (2008). Inhibitory effects of *Saururus chinensis* (Lour.) Baill on the development of atopic dermatitis-like skin lesions in NC/Nga mice. *Biological and Pharmaceutical Bulletin*.

[12] Ogawa K, Takeuchi M, Nakamura N (2005). Immunological effects of partially hydrolyzed arabinoxylan from corn husk in mice. *Bioscience, Biotechnology and Biochemistry*.

[13] Samukawa K, Izumi Y, Shiota M (2012). Red ginseng inhibits scratching behavior associated with atopic dermatitis in experimental animal models. *Journal of Pharmacological Sciences*.

[14] Tang H, Yang QP, Luo D (2011). Qingpeng ointment in the treatment of eczema: a multi-center, randomized, double-blind, placebo-controlled study. *Chinese Journal of Dermatology*.

[15] Proksch E, Brandner JM, Jensen J (2008). The skin: an indispensable barrier. *Experimental Dermatology*.

[16] Kezic S, Kemperman PMJH, Koster ES (2008). Loss-of-function mutations in the filaggrin gene lead to reduced level of natural moisturizing factor in the stratum corneum. *Journal of Investigative Dermatology*.

[17] Cork MJ, Danby SG, Vasilopoulos Y (2009). Epidermal barrier dysfunction in atopic dermatitis. *Journal of Investigative Dermatology*.

[18] Kim BE, Leung DYM, Boguniewicz M, Howell MD (2008). Loricrin and involucrin expression is down-regulated by Th2 cytokines through STAT-6. *Clinical Immunology*.

[19] Komatsu N, Saijoh K, Kuk C (2007). Human tissue kallikrein expression in the stratum corneum and serum of atopic dermatitis patients. *Experimental Dermatology*.

[20] Novak N, Leung DYM (2011). Advances in atopic dermatitis. *Current Opinion in Immunology*.

[21] Matsuoka H, Maki N, Yoshida S (2003). A mouse model of the atopic eczema/dermatitis syndrome by repeated application of a crude extract of house-dust mite *Dermatophagoides farinae*. *Allergy*.

[22] Li J, Roubeix C, Wang Y (2012). Therapeutic efficacy of trehalose eye drops for treatment of murine dry eye induced by an intelligently controlled environmental system. *Molecular Vision*.

[23] Livak KJ, Schmittgen TD (2001). Analysis of relative gene expression data using real-time quantitative PCR and the 2-ΔΔCT method. *Methods*.

[24] Grewe M, Bruijnzeel-Koomen CAFM, Schöpf E (1998). A role for Th1 and Th2 cells in the immunopathogenesis of atopic dermatitis. *Immunology Today*.

[25] Leung DYM, Boguniewicz M, Howell MD, Nomura I, Hamid QA (2004). New insights into atopic dermatitis. *Journal of Clinical Investigation*.

[26] Boguniewicz M, Leung DYM (2011). Atopic dermatitis: a disease of altered skin barrier and immune dysregulation. *Immunological Reviews*.

[27] Yuan-Yuan L, Lin-Feng L (2013). Inhibitory effect of Qingpeng ointment on experimental atopic dermatitis in mice. *Chinese Journal of Dermatology*.

[28] Laan MP, Koning H, Baert MRM (1998). Levels of soluble intercellular adhesion molecule-1, soluble E-selectin, tumor necrosis factor-*α*, and soluble tumor necrosis factor receptor p55 and p75 in atopic children. *Allergy*.

[29] Behniafard N, Gharagozlou M, Farhadi E (2012). TNF-*α* single nucleotide polymorphisms in atopic dermatitis. *European Cytokine Network*.

[30] Reynolds JM, Angkasekwinai P, Dong C (2010). IL-17 family member cytokines: regulation and function in innate immunity. *Cytokine and Growth Factor Reviews*.

[31] Hamada H, Garcia-Hernandez MDLL, Reome JB (2009). Tc17, a unique subset of CD8 T cells that can protect against lethal influenza challenge. *Journal of Immunology*.

[32] Martin-Orozco N, Muranski P, Chung Y (2009). T helper 17 cells promote cytotoxic T cell activation in tumor immunity. *Immunity*.

[33] Huber M, Heink S, Grothe H (2009). Th17-like developmental process leads to CD8^+^ Tc17 cells with reduced cytotoxic activity. *European Journal of Immunology*.

[34] Milner JD (2011). IL-17 producing cells in host defense and atopy. *Current Opinion in Immunology*.

[35] Ma D, Zhu X, Zhao P (2008). Profile of Th17 cytokines (IL-17, TGF-*β*, IL-6) and Th1 cytokine (IFN-*γ*) in patients with immune thrombocytopenic purpura. *Annals of Hematology*.

[36] Wong CK, Lit LCW, Tam LS, Li EKM, Wong PTY, Lam CWK (2008). Hyperproduction of IL-23 and IL-17 in patients with systemic lupus erythematosus: implications for Th17-mediated inflammation in auto-immunity. *Clinical Immunology*.

[37] Kawakami T, Ando T, Kimura M, Wilson BS, Kawakami Y (2009). Mast cells in atopic dermatitis. *Current Opinion in Immunology*.

[38] Liu FT, Goodarzi H, Chen HY (2011). IgE, mast cells, and eosinophils in atopic dermatitis. *Clinical Reviews in Allergy and Immunology*.

[39] Groneberg DA, Bester C, Grützkau A (2005). Mast cells and vasculature in atopic dermatitis—potential stimulus of neoangiogenesis. *Allergy*.

[40] Zhao L, Jin H, She R (2006). A rodent model for allergic dermatitis induced by flea antigens. *Veterinary Immunology and Immunopathology*.

[41] Kanbe T, Soma Y, Kawa Y, Kashima M, Mizoguchi M (2001). Serum levels of soluble stem cell factor and soluble KIT are elevated in patients with atopic dermatitis and correlate with the disease severity. *The British Journal of Dermatology*.

[42] Elias PM, Hatano Y, Williams ML (2008). Basis for the barrier abnormality in atopic dermatitis: outside-inside-outside pathogenic mechanisms. *Journal of Allergy and Clinical Immunology*.

